# Surface‐Enforced Alignment of Reprogrammable Liquid Crystalline Elastomers

**DOI:** 10.1002/advs.202204003

**Published:** 2022-08-21

**Authors:** Tayler S. Hebner, Bruce E. Kirkpatrick, Kristi S. Anseth, Christopher N. Bowman, Timothy J. White

**Affiliations:** ^1^ Department of Chemical and Biological Engineering University of Colorado Boulder CO 80303 USA; ^2^ Medical Scientist Training Program University of Colorado Anschutz Medical Campus Aurora CO 80045 USA; ^3^ Materials Science and Engineering Program University of Colorado Boulder CO 80303 USA

**Keywords:** covalent adaptable networks, liquid crystalline elastomers, soft robotics, stimuli‐responsive polymers

## Abstract

Liquid crystalline elastomers (LCEs) are stimuli‐responsive materials capable of undergoing large deformations. The thermomechanical response of LCEs is attributable to the coupling of polymer network properties and disruption of order between liquid crystalline mesogens. Complex deformations have been realized in LCEs by either programming the nematic director via surface‐enforced alignment or localized mechanical deformation in materials incorporating dynamic covalent chemistries. Here, the preparation of LCEs via thiol‐Michael addition reaction is reported that are amenable to surface‐enforced alignment. Afforded by the thiol‐Michael addition reaction, dynamic covalent bonds are uniquely incorporated in chemistries subject to surface‐enforce alignment. Accordingly, LCEs prepared with complex director profiles are able to be programmed and reprogrammed by (re)activating the dynamic covalent chemistry to realize distinctive shape transformations.

## Graphical abstract

Complex actuation of liquid crystalline elastomers (LCEs) is realized in a distinctive materials chemistry that is amenable to surface‐enforced alignment that incorporates dynamic covalent chemistry. LCEs are first programmed via surface patterning and subsequently (re)programmed by dynamic bond exchange. The incorporation of dynamic bonds is enabled by the use of thiol‐Michael oligomerization.

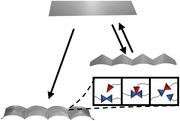

## 1 Introduction

Liquid crystalline elastomers (LCEs) have been extensively studied as stimuli‐responsive, shape‐changing materials. The ability of these materials to actuate controllably arises from the incorporation of liquid crystalline mesogens within a polymer network.^[^ ^1^ ^]^ When the liquid crystalline mesogens undergo an order disruption in response to an applied stimulus, the polymer network connecting these moieties is strained.^[^ ^2^, ^3^, ^4^ ^]^ In order to control the macroscopic deformation of the LCE, the material must be programmed such that the nematic ordering of the liquid crystal mesogens results in a macroscopically anisotropic LCE.^[^ ^5^, ^6^ ^]^ Once programmed, the LCE will contract along the direction of alignment and expand in orthogonal directions when the nematic ordering is disrupted, e.g., as a result of a phase change.^[^ ^7^, ^8^ ^]^

Typically, the alignment of LCEs for shape change programming is done either by surface anchoring or mechanical deformation.^[^ ^9^, ^10^, ^11^, ^12^ ^]^ Notably, these two types of alignment methods differ in the sense that surface alignment relies on molecular interactions of the liquid crystalline molecules with the surface, while mechanical alignment relies on the anisotropic stretching of the polymer network chains. In both cases, liquid crystalline monomers are combined with non‐liquid crystalline components to control the crosslink density and, through a series of processing steps, create an elastomeric material that retains the programmed alignment for a targeted shape change.^[^ ^13^, ^14^, ^15^, ^16^ ^]^

In surface alignment, liquid crystalline monomers are capillary filled between glass slides that have been coated with an alignment layer. In simple cases, typically when uniaxial alignment is desired, the surface coating is rubbed with a cloth to introduce the desired directional bias.^[^ ^17^, ^18^, ^19^, ^20^ ^]^ In another approach, a photoresponsive surface coating is used, and alignment is programmed by exposure to patterns of polarized light.^[^ ^21^, ^22^ ^]^ The photopatterning approach is a straightforward route to prepare LCEs capable of complex actuation. Once monomers are filled into an alignment cell and aligned with the surface patterning, a crosslinking step is initiated, often by exposure to light, to fix the alignment pattern after removal of the LCE sample from the cell. To create patterned materials that are elastomeric, the crosslinking step is often preceded by an oligomerization step.^[^ ^23^ ^]^ The oligomerization step allows for linear polymer segments to form before crosslinking, giving lower crosslink density. As a result, the LCE has a lower modulus, often a lower T_NI_, and typically undergoes larger magnitude deformations than a material crosslinked without the oligomerization step.

For surface aligned LCEs, one of the most commonly studied monomer systems is composed of acrylate‐based liquid crystalline monomers with a primary amine as a comonomer, enabling two‐step processing via aza‐Michael oligomerization and subsequent photopolymerization of acrylate‐terminated oligomers.^[^ ^24^, ^25^, ^26^ ^]^ A similar approach using thiol‐Michael oligomerization and subsequent photopolymerization of acrylate‐terminated oligomers has been previously used to fabricate LCEs by mechanical alignment and additive manufacturing techniques.^[^ ^12^, ^27^, ^28^, ^29^, ^30^, ^31^ ^]^ In this approach, using dithiols as comonomers enables variation of the spacer length and properties when a thiol‐Michael reaction is used to generate linear chains in the polymer before crosslinking.^[^ ^27^, ^32^ ^]^ To our knowledge, no prior study has detailed the preparation of LCEs amenable to surface‐enforced alignment based on thiol‐Michael addition reactions.

In addition to providing tunability in material properties by introducing variable length dithiol spacers, the thiol‐Michael oligomerization approach also provides the opportunity for introducing additional functional groups into the main chain of the network. Of particular interest is the incorporation of dynamic covalent bonds in LCEs.^[^ ^33^, ^34^ ^]^ By introducing dynamic covalent chemistry and thereby creating a covalent adaptable network (CAN), LCEs have been fabricated such that the orientation of the nematic director is (re)programmable simply by applying force and (re)activating the dynamic bond exchange by applying a stimulus.^[^ ^35^, ^36^, ^37^, ^38^, ^39^, ^40^, ^41^ ^]^ CANs have been implemented in LCEs as a method for both shape retention upon actuation and (re)programming of complex deformations.^[^ ^42^, ^43^, ^44^, ^45^ ^]^ Notably, numerous chemistries composed of acrylates and dithiols have been used in these dynamic systems, often taking advantage of the thiol‐Michael oligomerization step, but thus far none have been incorporated in surface‐aligned LCEs.^[^ ^46^, ^47^, ^48^, ^49^, ^50^, ^51^ ^]^ While these previous demonstrations utilize mechanical alignment, allowing for assessment of dynamic bond programming in a wider range of film thicknesses than can be achieved with surface enforced alignment (limited to ≈50 µm),^[^ ^47^, ^52^ ^]^ the surface alignment approach enables much higher spatial resolution of directed mesogen orientation.

Here, we take advantage of both complexity of surface alignment and the (re)programmability of CANs in LCEs by fabricating surface aligned LCEs using thiol‐Michael oligomerization to generate acrylate‐terminated oligomers for subsequent crosslinking via photopolymerization. Combining these programming techniques allows for the design of actuation that may not be accessible by either individual technique. The thiol‐Michael oligomerization approach was amenable to surface alignment across a broad range of compositions, leading to a range of accessible material properties in LCEs fabricated by this technique. Distinct surface‐aligned director patterns were created using a diacrylate liquid crystalline monomer and a dithiol containing allyl sulfide dynamic bonds. We used these two orthogonal shape‐programming techniques to create LCEs with complex and reprogrammable actuations.

## 2 Results and Discussion

### 2.1 LCEs Prepared via Thiol‐Michael Addition Subject to Surface‐Enforced Alignment

**Figure 1:**
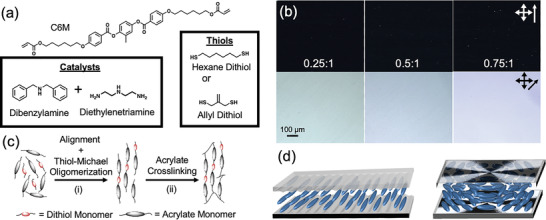
a) Chemical structures for the liquid crystalline diacrylate monomer, dithiol monomer, and Michael addition catalysts. b) The compositions are subjected to surface‐enforced alignment in cells prepared with planar (left) or photopatterned (right) director profiles. c) The two‐step reaction to prepare the LCE proceeds via (i) alignment and thiol‐Michael oligomerization and is followed by (ii) acrylate crosslinking. d) Polarized optical micrographs confirm the LCEs retain monodomain alignment for increasing thiol:acrylate ratios (left to right).

**Figure 2:**
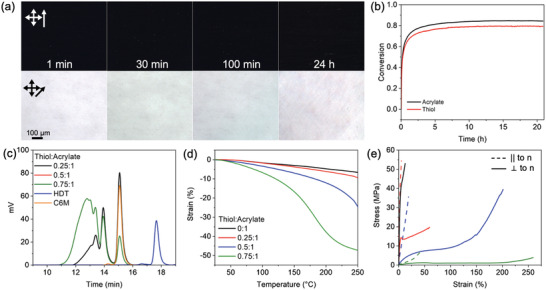
a) Polarized optical micrographs show alignment after thiol‐Michael oligomerization of a 0.75:1 thiol:acrylate mixture was carried out in the isotropic state for increasing amounts of time before aligning in the nematic state. b) Real‐time FTIR measurements during oligomerization confirm the reaction was complete after 22 h. c) GPC of the oligomers for the three thiol:acrylate ratios confirmed that increasing thiol concentration increased the oligomer molecular weight. d) Upon polymerization of the acrylate‐terminated oligomers (and residual thiol comonomer), the LCE exhibited thermomechanical strain when held in tension and heated (0.005 N,°C/min). Increasing thiol concentration increased the magnitude and rate of strain. e) Stress–strain response of LCEs in tension deformed parallel (dashed lines) and perpendicular (solid lines) to the nematic director (5%/min) for thiol:acrylate ratios of 0:1 (black), 0.25:1 (red), 0.5:1 (blue), and 0.75:1 (green).

Monodomain liquid crystalline elastomers (LCEs) were fabricated by surface‐enforced alignment. A thiol‐Michael reaction was used to incorporate dithiols between the diacrylate liquid crystalline monomers and form oligomers. To demonstrate the feasibility of alignment using this technique, LCEs were prepared using C6M and hexanedithiol (HDT) (**Figure** **1**a) in ratios of 0.25:1, 0.5:1 and 0.75:1 thiol:acrylate (molar ratios). The monomers were mixed with a combination of amine catalysts (dibenzylamine, 1 mol% and diethylenetriamine, 0.4 mol%). The mixtures were subsequently subjected to surface‐enforced alignment in cells prepared with rubbed coatings to induce a uniaxial monodomain orientation of the mesogens (Figure 1b). After filling in the isotropic state (90 °C), the samples were cooled into the nematic phase (65 °C) where they were held for 2 h before the temperature was increased to 80 °C for an additional 22 h to allow for the thiol‐Michael reaction to proceed in the aligned state (Figure 1c,i). The conversion data in **Figure** **2**b and Figures S1 and S4 (Supporting Information) indicate that this time period was sufficient for the thiol‐Michael reaction to go to completion. In the oligomerization step, while dibenzylamine was used as the primary catalyst, diethylenetriamine (DETA) was included to function as both a catalyst and to stabilize the retention of liquid crystallinity. This stability arises from introducing a small number of crosslinks via aza‐Michael addition and preventing crystallization of the linear oligomers. Control experiments presented in Figure S1 (Supporting Information) illustrate that DETA inclusion prevents crystallization of the oligomers that hinders further conversion of monomers in the thiol‐Michael reaction. Figure S1 (Supporting Information) also includes kinetic data using triethylamine instead of dibenzylamine, demonstrating that other common amine catalysts are amenable to this approach. After oligomerization was complete, the acrylate‐terminated oligomers were crosslinked by photoinitiated polymerization (Figure 1c,ii). The strong birefringence in the polarized optical micrographs of Figure 1d is indicative of the defect‐free alignment in the resulting monodomain LCEs. The alignment in each LCE was further confirmed with WAXS diffraction patterns (Figure S2, Supporting Information) with the corresponding order parameters presented in **Table** **1**.

**Table 1:** Summary of material properties and thermomechanical response for LCEs prepared by thiol‐Michael oligomerization

Thiol:acrylate	0:1	0.25:1	0.5:1	0.75:1
Order parameter^a)^	0.26	0.39	0.45	0.47
Strain at 250 °C [%]^b)^	6	9	24	47
Max strain rate [%/°C]^c)^	0.04	0.1	0.5	0.5
Parallel modulus [MPa]^d)^	1760 ± 30	780 ± 190	160 ± 190	17 ± 9
Perpendicular modulus [MPa]^e)^	945 ± 130	190 ± 20	40 ± 5	6 ± 1
*T*_g_ [°C]^(^ ^d)^	50.2	37.1	15.6	‐3.5

^a)^ Herman's orientation parameter calculated from WAXS diffraction patterns (Figure S2, Supporting Information)

^b)^ Calculated as maximum derivative of thermomechanical strain generation (temp ramp 5 °C min^‐1^) in the range of 25–250 °C

^c)^ Measured as maximum strain at 250 °C when temperature was ramped from 25 to 250 °C at 5 °C min^‐1^

^d)^ Tensile pull 5% min^‐1^, modulus calculated in linear strain regime (2–4%)

^e)^ Measured using midpoint in second DSC heating cycle (5 °C min^‐1^, Figure S12, Supporting Information).

Given the seemingly robust amenability of thiol‐Michael reaction mixtures to surface alignment across a broad range of thiol:acrylate compositions, we next investigated the effects of oligomerization extent on surface enforced alignment. Again, monodomain orientation was enforced in alignment cells, here filled with the 0.75:1 thiol:acrylate mixture (HDT and C6M). The samples were held in the isotropic state (90 °C) within the alignment cell for either 1 min, 30 min, 100 min, or 24 h before the temperature was reduced to transition the composition to the nematic state (65°). Despite the expected variation in molecular weight of the oligomers, strong and defect‐free birefringent patterns were evident for all four time points as seen in Figure 2a. Other mixtures, such as 0.25:1 and 0.5:1 thiol:acrylate, showed analogous results (Figure S3, Supporting Information). The reaction kinetics of the thiol‐Michael oligomerization step were monitored by real‐time FTIR measurements taken while the compositions were held at 90 °C (isotropic state). Shown in Figure 2b for the 0.75:1 thiol:acrylate mixture, the four time points in Figure 2a span from start to completion of the thiol‐Michael addition reaction (see Figure S4, Supporting Information) for 0.25:1 and 0.5:1 thiol:acrylate compositions). Therefore, we conclude that the oligomerization extent does not impact the ability to surface align these materials before crosslinking of acrylates, likely because entanglements between oligomers are not prominent enough to interfere with reorientation of the chains to the aligned state.

### 2.2 Material Properties of LCEs Prepared by Thiol‐Michael Addition

The oligomer size produced by various ratios of thiol:acrylate (C6M and HDT) was compared as seen in Figure 2c. As expected, increasing the thiol concentration also increases the final oligomer molecular weight. Accordingly, upon photoinitiated polymerization, the material properties of the LCEs prepared from these precursors strongly depended on the thiol concentration due to the significant variation in the molecular weight between crosslinks that results from the different oligomer molecular weights. Specifically, Figure 2d illustrates that increased thiol concentration resulted in larger thermomechanical strain generation. Figure 2e indicates that increasing thiol concentration softened the LCE. Both the increase in strain generation and softening are attributed to polymerization of higher molecular weight oligomers producing an LCE with a lower crosslink density and storage modulus. Maximum thermomechanical strain, maximum strain rate, elastic moduli parallel and perpendicular to the nematic director, and *T*_g_ for 0:1, 0.25:1, 0.5:1, and 0.75:1 thiol:acrylate compositions are summarized in Table 1. LCEs with thiol:acrylate ratios greater than 0.75:1 were prepared but not robust enough to allow for characterization with equivalent methods.

### 2.3 Reprogramming LCEs by Incorporating Dynamic Covalent Chemistry

The preparation of surface‐aligned LCEs via thiol‐Michael oligomerization enabled the introduction of dynamic covalent chemistry. As such, we replaced hexane dithiol from the initial compositions with allyl dithiol (ADT, Figure 1a) at a ratio of 0.5:1 thiol:acrylate to prepare LCEs that were elastomeric yet robust enough to fold for secondary programming. The LCEs were prepared via a visible light photoinitiation of the allyl‐sulfide containing diacrylate oligomers (Speedcure VLT, 525 nm exposure). A second UV photoinitiator (Omnirad 819, 365 nm exposure) was also included for subsequent photoinitiation of the allyl sulfide bond exchange. The monomer mixture was oligomerized and crosslinked in an alignment cell programmed with a director profile described as a +1 topological defect (Figure 1b).

**Figure 3:**
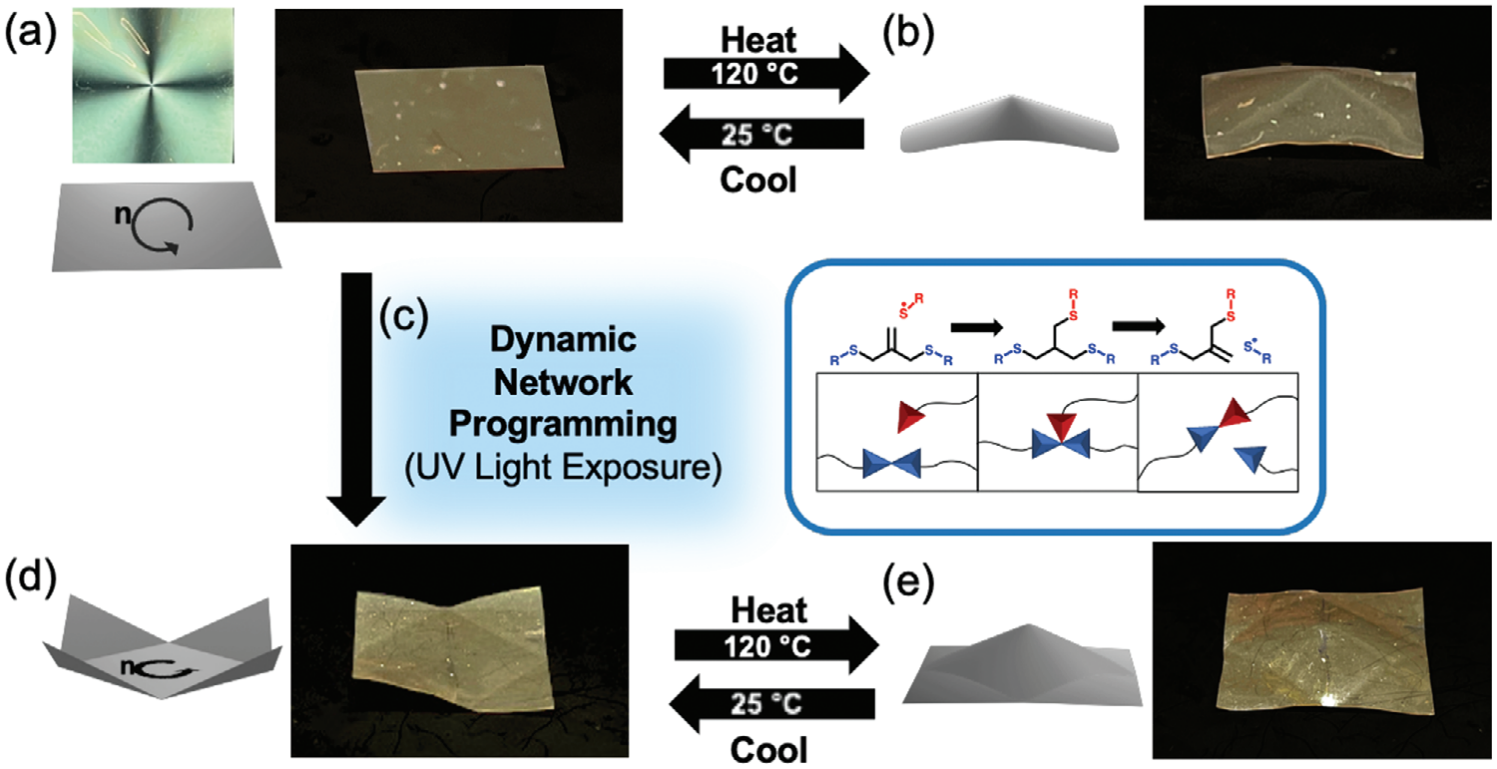
a) An LCE is prepared with a director profile described as *a* +1 topological defect. After preparation, the LCE is a flat sheet. b) The LCE deforms into a cone upon heating to 120 °C. c) The corners of the LCE are then folded and the material is exposed to UV light, activating dynamic bond exchange of allyl sulfide groups. After secondary programming, d) the LCE retains the overall +1 defect pattern with the folded pattern and upon heating to e) 120 °C deforms into a cone with a perimeter defined by the folded pattern and corners that remain flat.

The LCE was harvested from the cell as a flat sheet (**Figure** **3**a). Upon heating to 120 *°*C, the LCE morphed into a cone (Figure 3b) before returning to the flat state on cooling to room temperature (Video S1, (Supporting Information). The LCE was then subjected to shape programming using the allyl sulfide‐based dynamic bond exchange. The corners of the LCE sheet were folded (Figure S5, Supporting Information) and while deformed, the material was exposed to UV light to induce allyl sulfide bond exchange within the network (reaction mechanism illustrated in Figure 3c). Stress relaxation measurements confirming bond exchange during UV exposure are presented in Figure S6 (Supporting Information), confirming that the 10 min exposure was sufficient for the bond exchange to relax the material to the greatest extent possible with this network configuration and film thickness. The bond exchange further programmed the LCE to retain the folded structure at room temperature (Figure 3d). Control experiments in LCEs prepared by thiol‐Michael oligomerization of HDT and C6M or by radical‐mediated photopolymerization of allyl dithiol and C6M (e.g., primarily leaving dithiols as pendant groups^[^ ^53^ ^]^) confirm the secondary shape fixing was only observed when the LCE was fabricated via thiol‐Michael oligomerization of allyl dithiol and C6M (Figures S7 and S8, Supporting Information). The programmed LCE deformed upon heating to form the structure shown in Figure 3e. The observed shape is a result of the LCE continuing to respond to produce the out‐of‐plane cone in the central region with the secondary programming distinguishing a square boundary around the cone as the corners flattened against the hot surface. Again, the deformation of the programmed LCE was reversible upon heating and cooling (Video S2, Supporting Information). In this demonstration, we confirmed that orthogonal programming steps generate complex actuation via patterning of both liquid crystalline packing and polymer network configuration.

**Figure 4:**
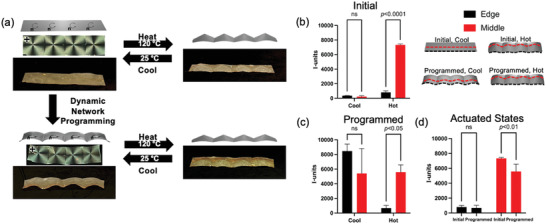
a) A patterned LCE containing dynamic covalent chemistry is prepared with four adjacent +1 topological defects. Dynamic network programming facilitates secondary reprogramming to alter the initial shape of the material while retaining the original actuated shape with four adjacent cones. The deformation of the LCE was analyzed in I‐units for profiles along the edge and middle of b) the initial cool and hot states, c) the cool and hot states after dynamic network programming, and (d) both actuated states. Data for I‐unit analyses are presented as mean ± standard deviation for *n* = 3 with two‐way ANOVA comparisons where ns indicates “not significant.”

In a second demonstration, we designed an LCE to have a distinct shape at room‐temperature based on dynamic bond programming, but retain the actuated state induced by nematic director programming. To fabricate the LCE, four +1 defects were first patterned adjacent to one another. Upon heating, the LCE transformed from flat to a shape defined by the formation of four adjacent cones (**Figure** **4**a and Video S3, Supporting Information). To assess the initial and actuated states, we quantified flatness across the LCE surface using I‐unit analysis. I‐units relate sinusoidal peak height (*H*) and the distance between peaks (*L*) in a single dimensionless parameter.^[^ ^54^ ^]^ In essence, the I‐unit metric compares the projected (flat) length of an angled surface to its true length, where 1000 I‐units imply that the true length can be attained by applying 1% strain to the projected value. Additional details on I‐unit calculations are provided in Figure S9 (Supporting Information). The flatness of the LCE upon preparation had no statistical difference when comparing measurements along the edge and middle of the adjacent +1 defect patterns (Figure 4b). As expected, the values confirm significant wave‐like deformation across the material in the central region while the edges remained relatively flat. The statistical differences between the actuated edge and middle measurements are consistent with the adjacent cone deformations that would be expected from an enforced director profile.

After characterizing the initial stimulus response, the LCE was deformed to introduce folds along the interface between each of the +1 defects. The allyl sulfide‐containing LCE was then exposed to UV light to induce dynamic bond exchange. After secondary programming, the initial shape of the LCE was generally flat with four adjacent curved regions. Upon heating, the LCE still deformed reversibly into adjacent cones (Figure 4a and Video S4, Supporting Information), illustrating that the dynamic bond exchange used in orthogonal programming selectively altered only the initial shape while retaining the deformed shape programmed by surface alignment. The shapes were also quantified by I‐units for the initial and actuated states after programming as shown in Figure 4c. In the initial cool state, the edge and middle measurements both indicated wave‐like deformation with no statistical significance between the two. In the deformed state, we observed the same trend in the difference between the edge and middle as in the LCE before dynamic bond programming, confirming the adjacent cone deformations had been retained. Notably, these measurements indicate that while the shape is maintained, the magnitude of the deformation in the actuated state was slightly decreased after the programming step (Figure 3d), potentially being attributable to a slight reduction in order.

**Figure 5:**
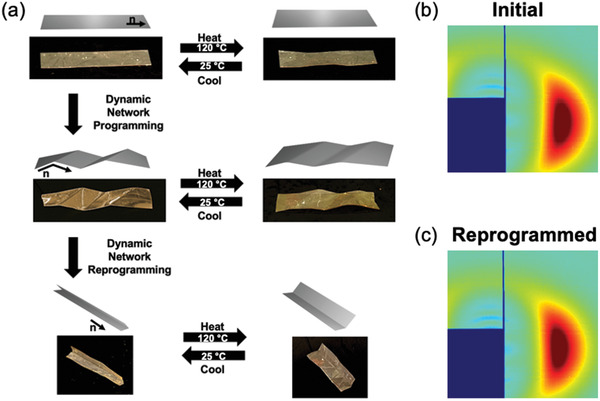
a) An LCE with a uniaxial surface patterned director is subjected to two sequential programming steps via dynamic bond exchange to realize distinct, reversible shape transformations. b) WAXS diffraction patterns confirm retention of orientation in the LCE before and c) after programming.

Using the incorporated dynamic covalent chemistry, the LCE was able to be programmed and reprogrammed. Since the dynamic bonds employed here undergo radical‐mediated exchange, the bond exchange step was repeated by introducing additional photoinitiator to the system. The (re)programming process is illustrated in **Figure** **5**a. Here, the LCE was first patterned with a uniaxial nematic director. Upon heating to 120 °C, the LCE exhibited reversible, linear contraction (Video S5, Supporting Information). The LCE was then folded and exposed to UV light to program diagonal ridges in the material. Upon reversible thermomechanical actuation, these sharp ridges became smooth (Video S6, Supporting Information). Subsequently, the material was programmed a second time, with additional photoinitiator being incorporated in the LCE via swelling in a DCM solution. After drying, the LCE was folded along its long axis and exposed to UV light (50 mW cm^‐2^, 10 min). After reprogramming, the folded LCE opened up along the fold like a hinge (Video S7, Supporting Information). Notably, even after two secondary programming steps, the LCE retained the initial surface programmed nematic director except along the fold lines (Figure S10, Supporting Information). The orientation parameter evident in the WAXS diffraction patterns collected before (Figure 5b) and after (Figure 5c) dynamic bond programming were 0.50 and 0.54, respectively.

## 3 Conclusion

LCEs were fabricated via thiol‐Michael oligomerization and subsequent photopolymerization of acrylate‐terminated oligomers within a surface alignment cell, resulting in monodomain LCEs with tunable mechanical properties. Allyl dithiol was introduced to realize LCEs amenable to surface‐enforced alignment that also contain dynamic covalent bonds. The surface aligned materials were subjected to secondary programming via folding and light‐activated dynamic bond exchange, resulting in complex deformations of the LCEs upon exposure to heat.

## 4 Experimental Section

### Fabrication of Alignment Cells

For alignment of uniaxial/twisted nematic materials, precleaned glass slides (Colorado Concepts) were coated with a solution of Elvamide in methanol. The surface of each coated slide was rubbed uniaxially with velvet. Cells were assembled by adhering two coated slides with Norland Optical Adhesive 68 containing 30 µm glass rod spacers. For uniaxial monodomain alignment, coated slides were adhered with anti‐parallel alignment of rubbing direction. For twisted nematic alignment, coated slides were adhered with 90° rotation of rubbing direction relative to one another.

For materials with +1 topological defects, precleaned glass slides (Colorado Concepts) were coated with a solution of brilliant yellow (Sigma) in THF (1 wt%). Coated slides were cured at 100 °C for 30 min before adhering of two slides together with Norland Optical Adhesive 68 containing 30 µm glass rod spacers. A 445 nm vertically polarized laser (5 mW cm^‐2^ was then used to pattern the cells with a +1 topological defect via exposure through a *q* = +½ waveplate (BEAM Co.) for 10 min per defect. Laser cut masks were used to cut the beam to a square perimeter of either 7 mm (small defects) or 15 mm (large defects).

### Synthesis of Liquid Crystalline Elastomers

Elastomers were fabricated using a combination of 1,4‐Bis‐[4‐(6‐acryloyloxyhexyloxy)benzoyloxy]‐2‐methylbenzene (C6M, Wilshire Technologies) with hexanedithiol (HDT, Sigma), hexylamine (Sigma) or allyl dithiol (ADT, synthesized according to previously documented methods,^[^ ^47^ ^]^ NMR in Figure S15, Supporting Information).

For materials using C6M and HDT, monomers were combined in molar ratios of 0.25:1, 0.5:1, and 0.75:1 thiol:acrylate along with 1 wt% Omnirad 819 as a photoinitiator. Monomers were heated to 150 °C and vortex mixed before adding dibenzylamine (1 mol%) and diethylenetriamine (0.4 mol%) as catalysts and subjecting the mixture to additional vortex mixing. The mixture was then capillary filled into a 30 µm alignment cell at 90 °C. For materials used in mechanical and actuation testing, the cell was moved to 65 °C immediately after filling and held at this temperature for 2 h, then temperature was increased to 80 °C for 22 h to allow for the thiol‐Michael reaction to proceed in the nematic state. For materials used in oligomerization time studies, individual cells containing the 0.75:1 thiol:acrylate composition were held at 90 °C (isotropic state) for 1, 30, 100 min, and 24 h after filling before moving to 65 °C (nematic state). For the materials moved to the nematic state after 1 min and 30 min, the temperature was held at 65 °C for 2 h before moving to 80 °C for the remainder of the 24 h oligomerization period. For the materials held isotropic for 100 min, the temperature was held at 65 °C for 20 min before moving to 80 °C for the remainder of the 24 h oligomerization period. The materials held isotropic for 24 h were moved to 65 °C for 10 min before moving to 80 °C for 5 additional minutes. After the 24 h oligomerization, all materials were exposed to 365 nm light (50 mW cm^‐2^) for 10 min while remaining at 80 °C to crosslink the materials via homopolymerization of excess acrylates.

For materials using C6M and ADT, monomers were combined in a ratio of 0.5:1 thiol:acrylate along with 1 wt% Omnirad 819 and 2 wt% Speedcure VLT (Sartomer) as photoinitiators. Monomers were heated to 150 °C and vortex mixed before adding dibenzylamine (1 mol%) and diethylenetriamine (0.4 mol%) as catalysts and subjecting the mixture to additional vortex mixing. The mixture was then capillary filled into a 30 µm alignment cell at 90 °C and moved to 65 °C immediately after filling. The cell was held at 65 °C temperature for 2 h, then at 80 °C for 22 h and subsequently exposed to 525 nm light (50 mW cm^‐2^) for 45 min. Polymer films were then removed from the alignment cells.

For materials using C6M and hexylamine, monomers were combined in molar ratios of 0.25:1, 0.5:1, and 0.75:1 amine:acrylate along with 1 wt% Omnirad 819 as a photoinitiator. C6M and photoinitiator were heated to 150 °C and vortex mixed before adding amine and subjecting the mixture to additional vortex mixing and heating at 90 °C. Melted mixtures were then capillary filled into 30 µm alignment cells at 90 °C. Cells were moved to 65 °C immediately after filling and held at this temperature for 24 h to allow for the aza‐Michael reaction to proceed before exposing to 365 nm light (50 mW cm^‐2^) for 10 min. Polymer films were then removed from the alignment cells.

### Thermomechanical Actuation

LCEs were cut into strips (≈2 mm × 12 mm × 0.03 mm) along the aligned axis and placed in tension (Discovery DMA 850, TA Instruments) at 0.005 N constant force. Thermomechanical strain response was measured as temperature was increased from 25 °C to 250 °C at 5 °C min^‐1^.

For visual representation of actuation, patterned LCEs were placed on a hot plate at 120 °C. Images were taken before and after thermal deformation and videos were recorded upon heating and cooling of the material. Topographical representations of deformed LCEs in their final state were imaged using a Keyence VR‐3200 optical profilometer.

### Shape Programming Using Dynamic Covalent Bonds

For programming at room temperature, LCEs were folded with desired shape and exposed to 365 nm light (50 mW cm^‐2^) for 10 min. For programming in the actuated state, the LCE was placed on a hot plate at 120°C and allowed to deform until it reached a stable state. The material was then exposed to 365 nm light (50 mW cm^‐2^) for 10 min while the material remained at 120 °C.

For second programming cycles, materials were allowed to swell in a solution of DCM with Omnirad 819 (0.67 mg mL^‐1^) for 24 h. After swelling, materials were allowed to dry for 24 h before programming as previously described.

### Tensile Testing

Tensile experiments were conducted by dynamic mechanical analysis (RSA‐G2, TA Instruments) on LCE strips cut either perpendicular or parallel to the nematic director. The strain was applied at 5% min^‐1^. The moduli of the LCEs were taken in the linear strain regime (2–4% strain).

### Gel Permeation Chromatography

Gel permeation chromatography (GPC) was performed on an Ecosec HLC‐8320 GPC with chloroform as a solvent at a flow rate of 0.5 mL min^‐1^ through a 15 cm TSKgel Super HM‐N column. Samples were prepared in chloroform solutions at a concentration of 1.5 mg mL^‐1^ and were filtered through a 0.45 µm syringe filter before measurement. Refractive index detector data was used for analysis.

### Polarized Optical Microscopy

Polarized optical micrographs were captured using a Nikon polarizing microscope.

### Real‐Time Fourier Transform Infrared Spectroscopy

Real‐time FTIR was performed using a Nicolet iS50 and a custom heating accessory. Before measurement, monomeric mixtures were melted and prepared as described previously but without photoinitiator included. A sample of each melted mixture was sandwiched between NaCl salt plates and placed on the heating stage at 90 °C. Absorption spectra were collected every 1 min for 20 h. Changes in the area of thiol (2550–2600 cm^‐1^) and acrylate (800–820 cm^‐1^) peaks were monitored and conversion was calculated using the difference between the initial peak area and peak area at each time point.

### Differential Scanning Calorimetry

*T*_g_ of each LCE was measured using differential scanning calorimetry (Discovery DSC 2500, TA Instruments). Data are reported from the second heating cycles performed at a ramp rate of 5 °C min^‐1^.

### Wide Angle X‐Ray Scattering

Static WAXS experiments were collected on beamline 11‐BM Complex Materials Scattering at the National Synchrotron Light Source II at Brookhaven National Laboratory. Patterns were acquired on samples using 13.5 keV energy and ten second exposures. Data reduction and order parameter calculations were performed in Igor using the Nika analytical package and through a custom script in Matlab, respectively.

### Statistical Analysis

For I‐unit calculations, length measurements were determined by either profilometry or image analysis in FIJI. These values were used in the equations shown in Figure S9 (Supporting Information) to calculate the dimensionless I‐units. Each length value (e.g., the height and width of an edge) was measured with *n* = 3 across the repeated topological defects in each film. Two‐way analysis of variance (ANOVA) with Tukey's multiple comparisons test was performed in GraphPad PRISM on pooled replicate I‐unit values for each condition, grouped by location (edge/middle) and actuation state (cool/hot) (with the exception of Figure S9E, Supporting Information which shows the difference between actuated and unactuated states and was grouped by analysis method & location). Data and error bars are presented as mean ± standard deviation.

For elastic moduli, data are presented as mean ± standard deviation with *n* = 3.

## Conflict of Interest

The authors declare no conflict of interest.

## Supporting information

Supporting Information

Supplemental Video 1

Supplemental Video 2

Supplemental Video 3

Supplemental Video 4

Supplemental Video 5

Supplemental Video 6

Supplemental Video 7

Supplemental Video 8

Supplemental Video 9

## Data Availability

The data that support the findings of this study are available from the corresponding author upon reasonable request.
