# Supplementary material for: Surface‐Enforced Alignment of Reprogrammable Liquid Crystalline Elastomers
Source: Adv Sci (Weinh). 2022 Aug 21;9(29):2204003. doi: 10.1002/advs.202204003 (PMC9561760; doi:10.1002/advs.202204003)
Supplement: Supplementary file 1 — Supporting Information [file ADVS-9-2204003-s006.pdf]

ADVANCED SCIENCE
Open Access

Supporting Information

for *Adv. Sci.*, DOI 10.1002/advs.202204003

Surface-Enforced Alignment of Reprogrammable Liquid Crystalline Elastomers

*Tayler S. Hebner, Bruce E. Kirkpatrick, Kristi S. Anseth, Christopher N. Bowman and Timothy J. White**

Supporting Information

Orthogonal Programming of Shape Change in Liquid Crystalline Elastomers with Tunable Mechanical Properties

*Tayler S. Hebner, Bruce E. Kirkpatrick, Kristi S. Anseth, Christopher N. Bowman, and Timothy J. White**

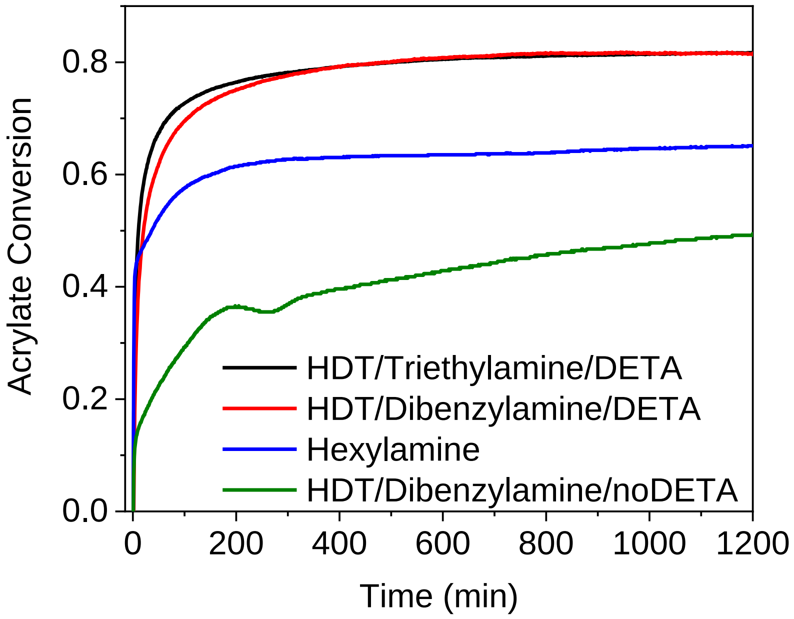

Figure S1. Real-time fourier infrared transform spectroscopy showing conversion of acrylates during oligomerization at 90°C for 0.75:1 thiol:acrylate with different catalysts and comparison to aza-Michael oligomerization with 0.75:1 amine:acrylate. Conversion measured using acrylate peak centered at 810 cm^{-1}

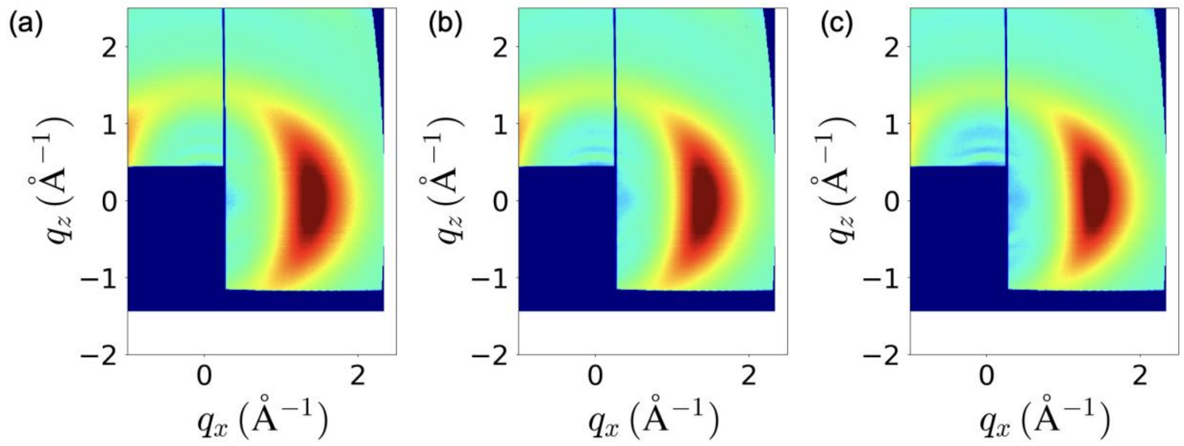

Figure S2. WAXS diffraction patterns for surface aligned LCEs with (a) 0.25:1, (b) 0.5:1, and (c) 0.75:1 thiol:acrylate (C6M, HDT).

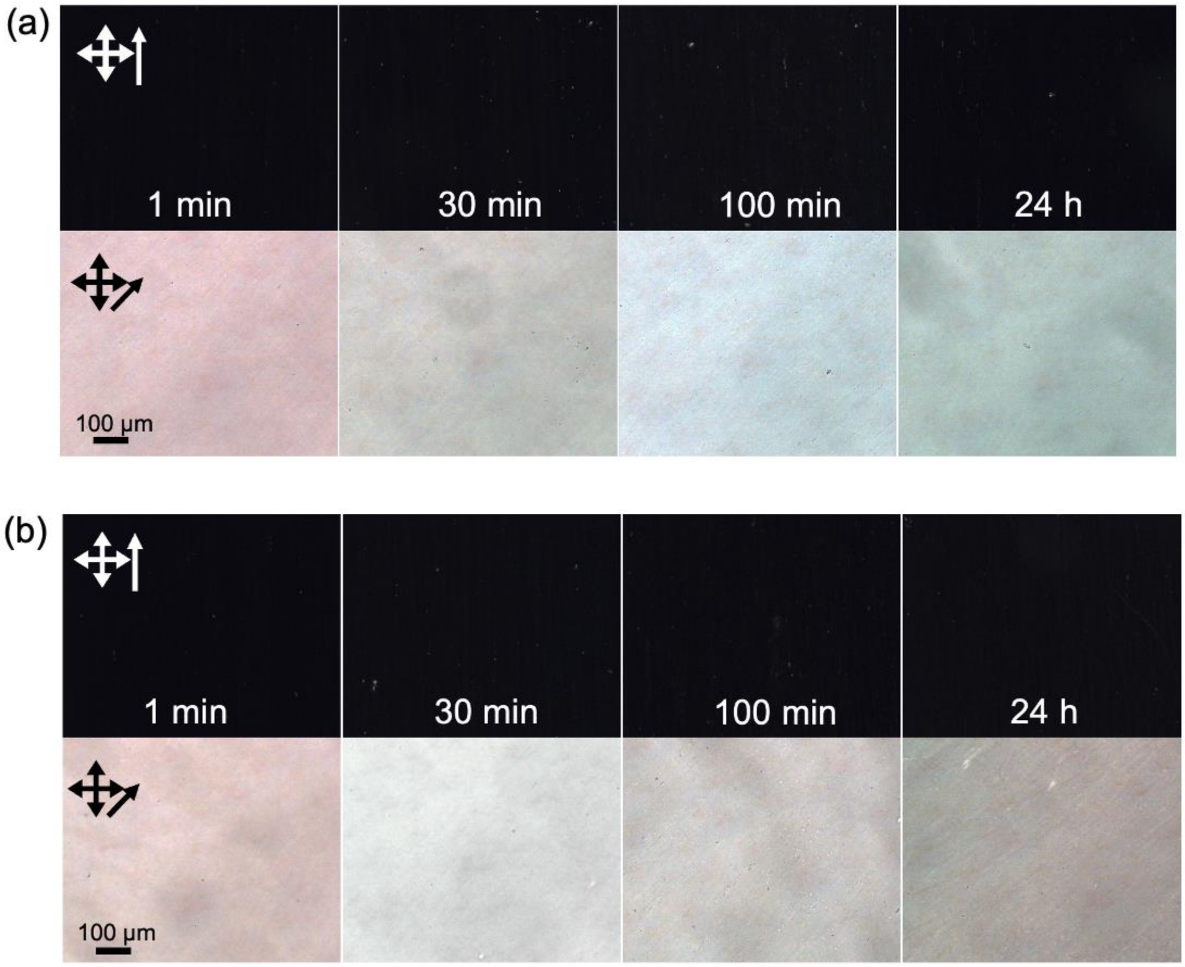

Figure S3. Polarized optical micrographs showing alignment as filled alignment cells are cooled to the nematic state after oligomerizing isotropic (90 °C) for increasing amounts of time for (a) 0.25:1 and (b) 0.5:1 thiol:acrylate using hexanedithiol and C6M

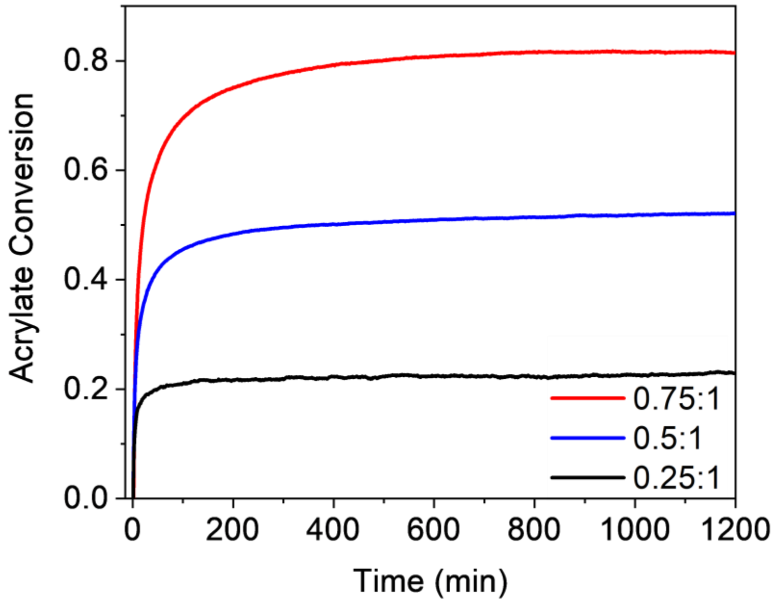

Figure S4. Real-time fourier infrared transform spectroscopy showing conversion of acrylates during oligomerization at 90°C for three different thiol:acrylate ratios with dibenzylamine/DETA used as the catalyst combination for all compositions. Conversion measured using acrylate peak centered at 810 cm^{-1}

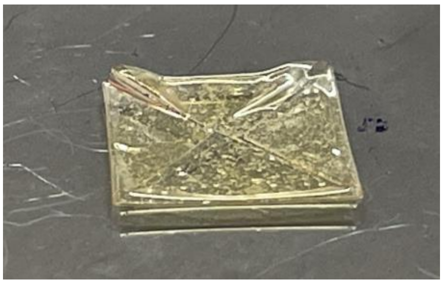

Figure S5. Folded state of an LCE with a +1 topological defect for programming by folding of corners to the center of the square.

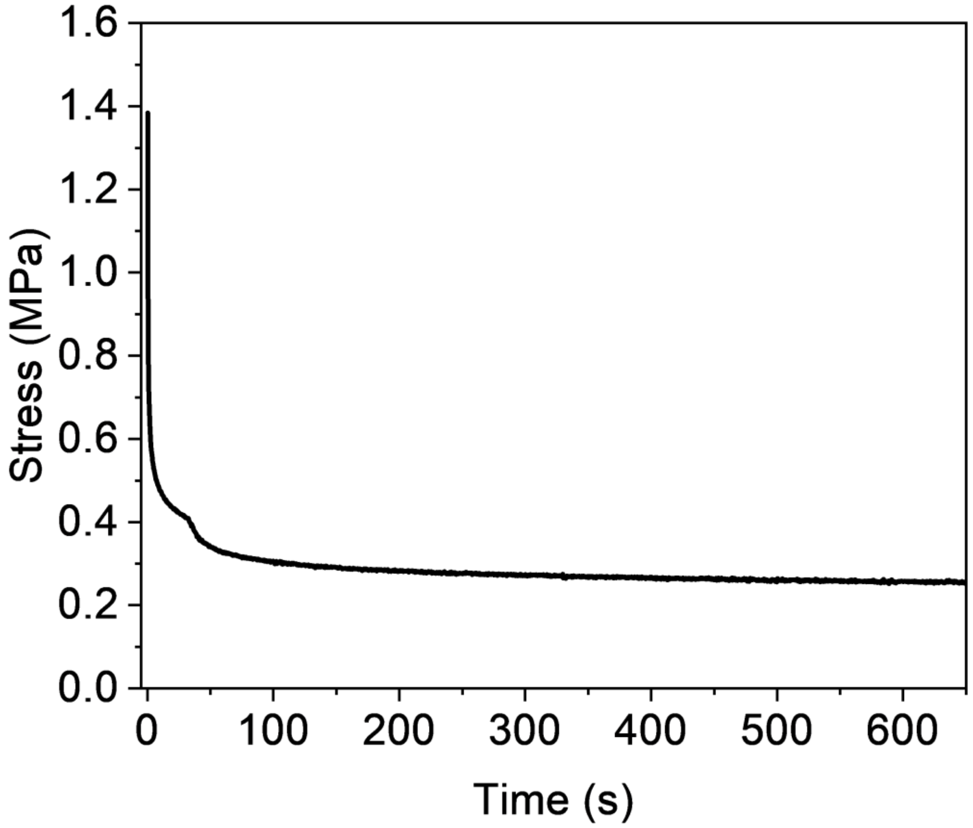

Figure S6. Stress relaxation measurements for 0.5:1 thiol:acrylate (allyl dithiol, C6M). Material subjected to 5% static strain applied, light exposure (50 mW/cm^2) at 30 seconds. Initial relaxation is seen due to liquid crystalline properties with additional relaxation from bond reorientation.

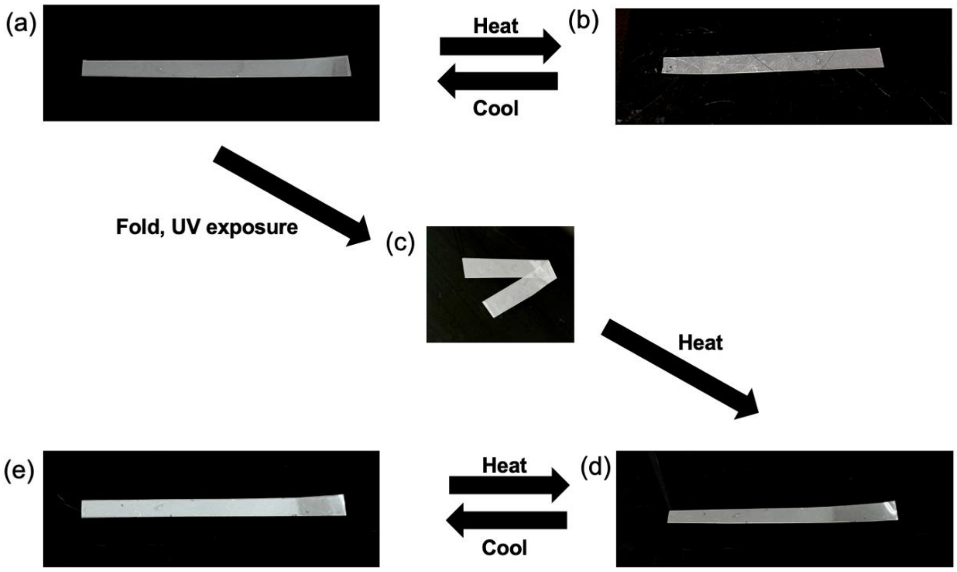

Figure S7. An LCE fabricated by radical-mediated thiol-acrylate photopolymerization using C6M and allyl dithiol in a 0.75:1 thiol:acrylate ratio in (a) the initial state, (b) the actuated state (120°C), (c) when folded and exposed to UV light, (d) when heated after UV exposure, showing no retention of the programmed fold, and (e) after cooling, showing that the LCE returns to the initial flat state and undergoes reversible linear contraction with no retention of the programmed fold

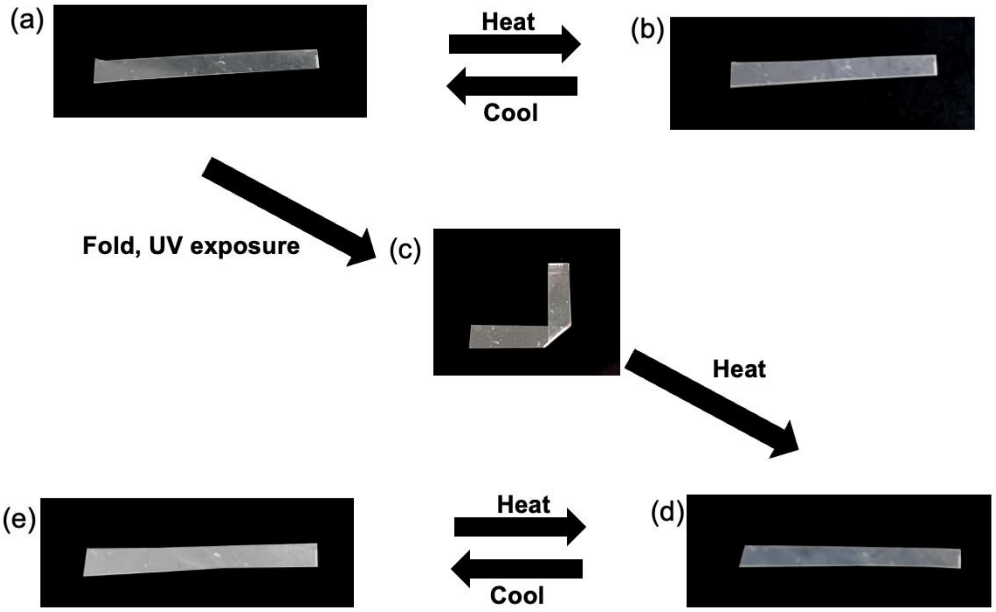

Figure S8. An LCE fabricated using C6M and hexanedithiol in a 0.5:1 thiol:acrylate ratio in (a) the initial state, (b) the actuated state (120°C), (c) when folded and exposed to UV light, (d) when heated after UV exposure, showing no retention of the programmed fold, and (e) after cooling, showing that the LCE returns to the initial flat state and undergoes reversible linear contraction with no retention of the programmed fold**.**

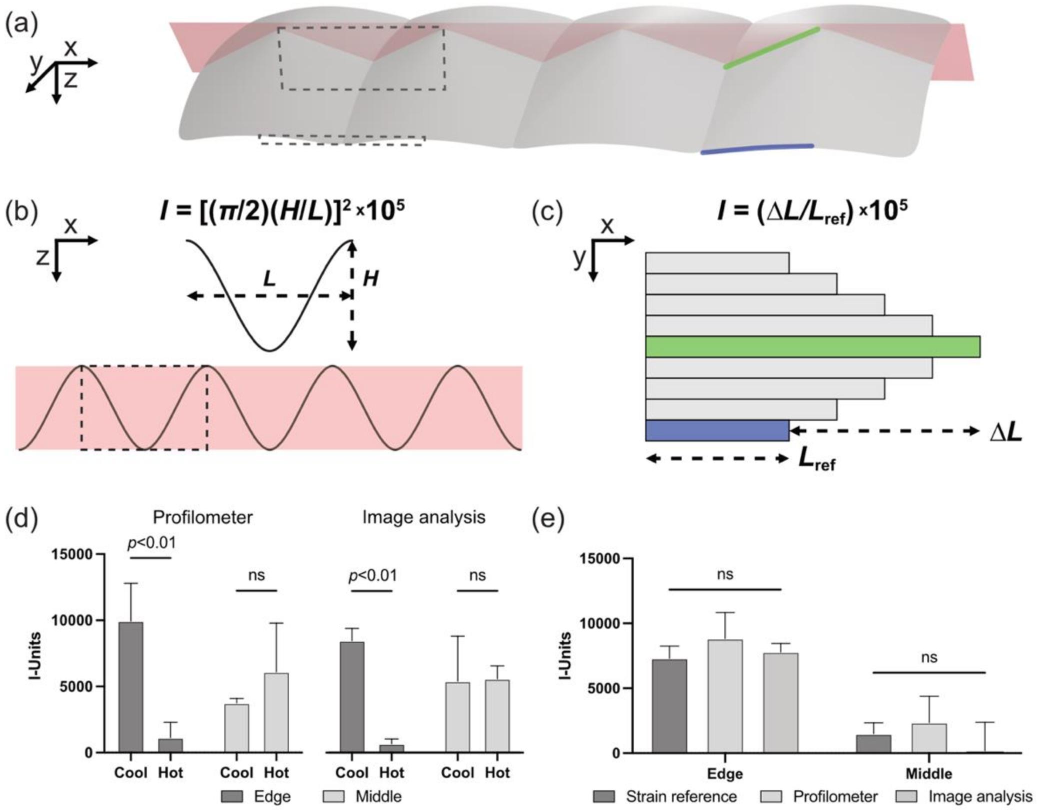

Figure S9. Basis of I-unit analysis. (a) Schematic of the actuated LCE shown in Figure 4a, with dashed lines highlighting 'edge' and 'middle' regions in the center *x-z* cross-section (red plane) for use in the sinusoidal I-unit equation. Blue and green lines represent reference and extended lengths along the LCE surface for use in the strain reference I-unit equation. (b) A sinusoidal representation of the LCE topology in *x-z* allows for calculation of I-units by relating the amplitude and wavelength of the sine function. (c) Approximating the actuated surface as a series of strained strips in *x-y* enables comparison between the shortest (blue) and longest strips (green) for calculation of I-units as a straightforward strain value. (d) Image analysis and profilometry data for the programmed LCE film in Figure 4c demonstrating that both datasets, using the sinusoidal representation, yield similar measured I-unit values and result in identical statistical comparisons. (e) By taking the difference between initial (cool) and actuated (hot) states, the strain reference value can be calculated from I-unit measurements made with the sinusoidal calculation. Profilometry data was used to calculate the surface length along the edge and center (blue and green lines in panels (a) and (c), respectively) of +1 topological defects on LCE films post-dynamic network reprogramming for I-unit analysis. Comparing these 'strain reference' values to those measured by the sinusoidal calculation on profilometry and imaging data shows no significant differences between any of the datasets or mathematical approaches. Data for I-unit analyses are presented as mean ± standard deviation for n = 3 with 2-way ANOVA comparisons where ns indicates "not significant".

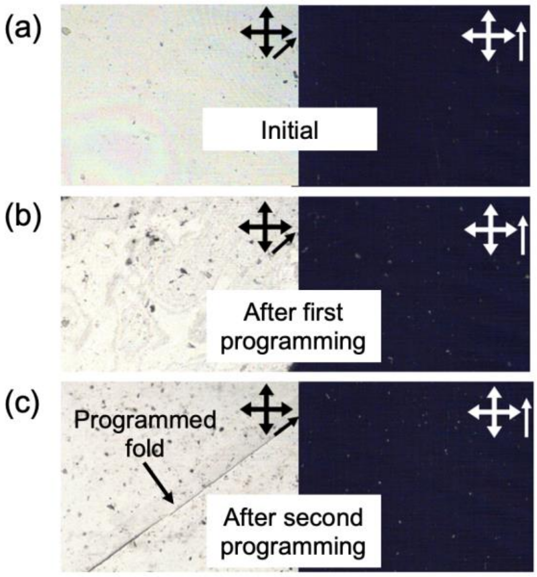

Figure S10. Polarized optical migrographs of an LCE fabricated with C6M and allyl dithiol (0.75:1 thiol:acrylate) after (a) initial surface alignment, (b) after exposure to UV light (10 min, 50 mW/cm^2) for dynamic bond exchange, and (c) after a second exposure to UV light (10 min, 50 mW/cm^2) with the region where the LCE was folded for programming showing no birefringence, confirming that the LCEs can be programmed with dynamic bond exchange and retain the surface programming in all regions except those that are folded/stretched upon UV exposure.

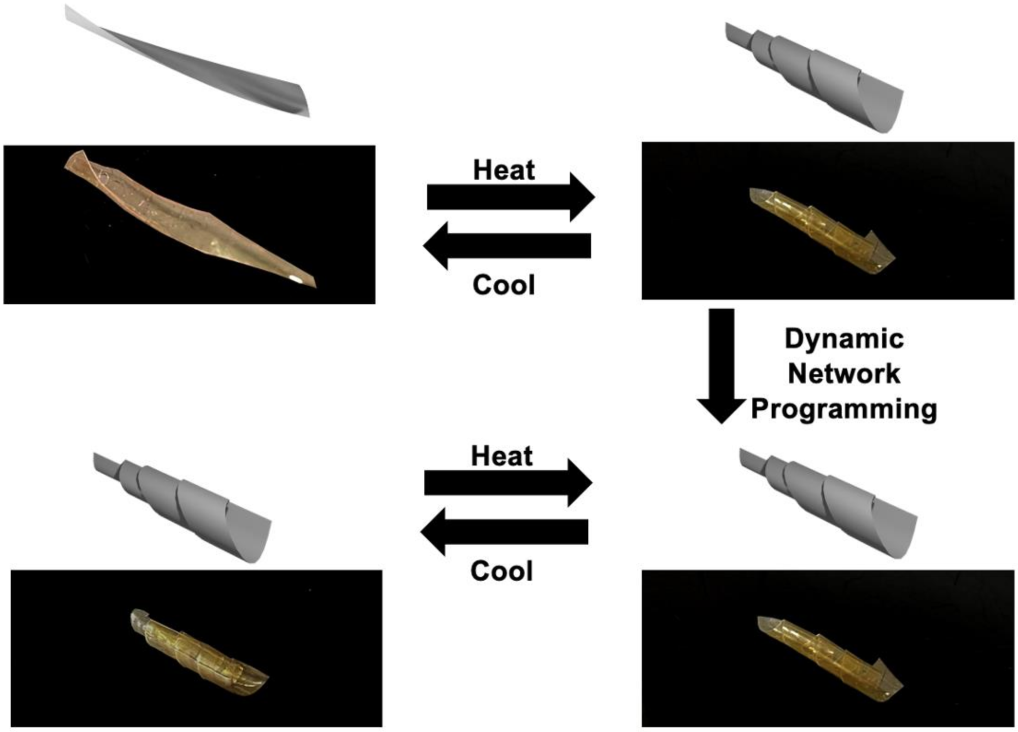

Figure S11. An LCE with twisted nematic director profile undergoes deformation to a curled shape with heating and retains this shape permanently with dynamic bond programming while actuated.

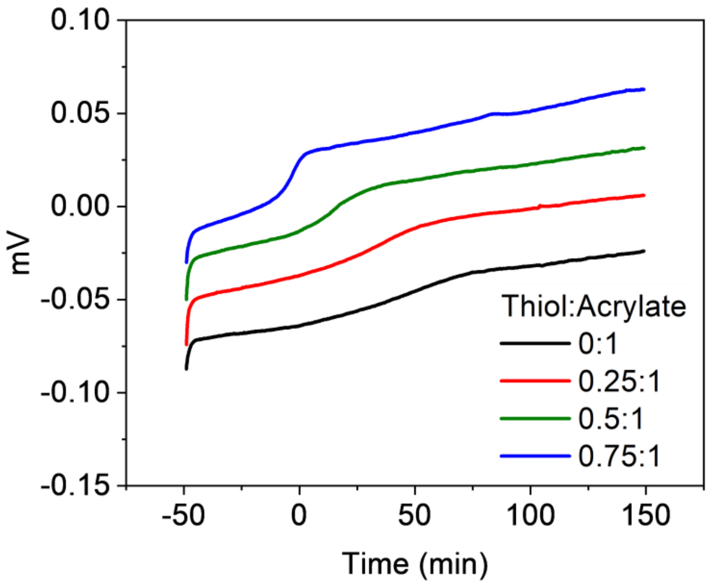

Figure S12. Differential scanning calorimetry traces (2nd heating, 5°C/min) for liquid crystalline polymers fabricated using C6M and HDT with various thiol:acrylate ratios.

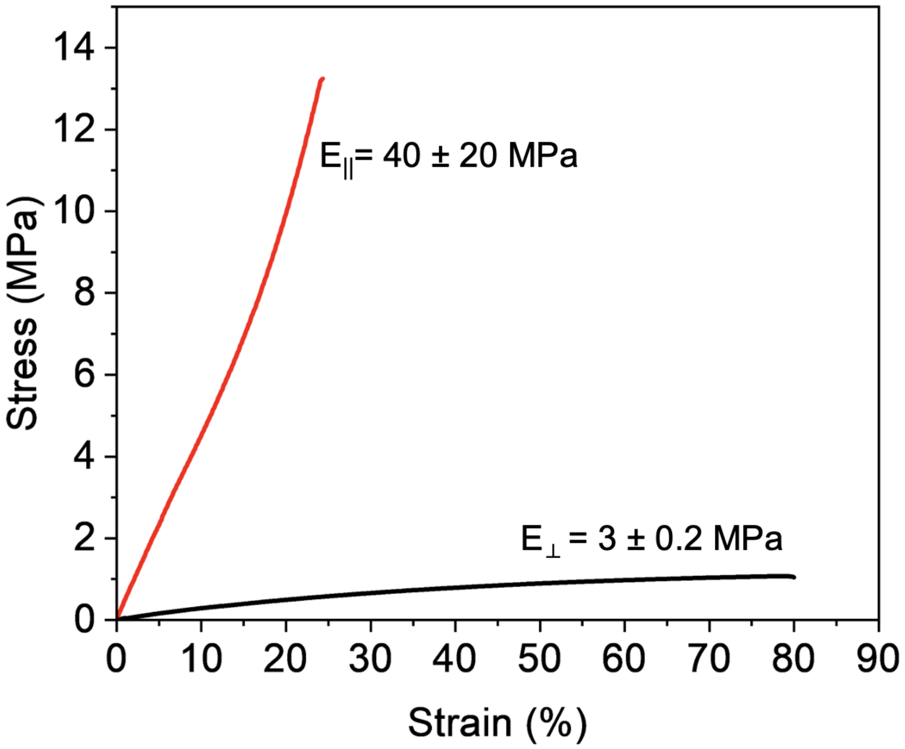

Figure S13. Tensile measurements for 0.5:1 thiol:acrylate (Allyl Dithiol, C6M). Materials were pulled at 5 %/min parallel or perpendicular to the nematic director. Modulus values were calculated in the linear strain regime (2-4 %)

(a)

(b)

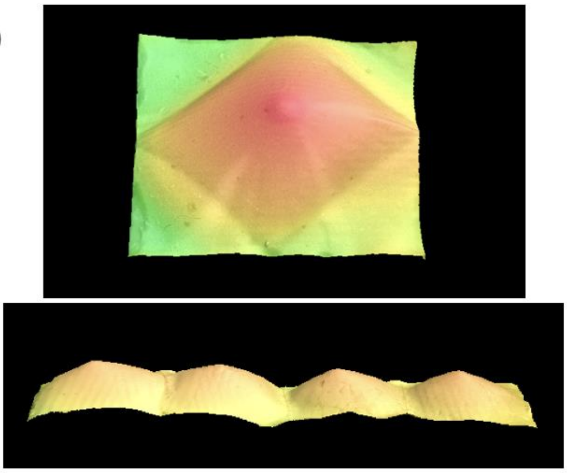

Figure S14. Topographical representations of LCEs with (a) a single +1 defect and (b) four adjacent +1 defects in the actuated state after dynamic bond programming

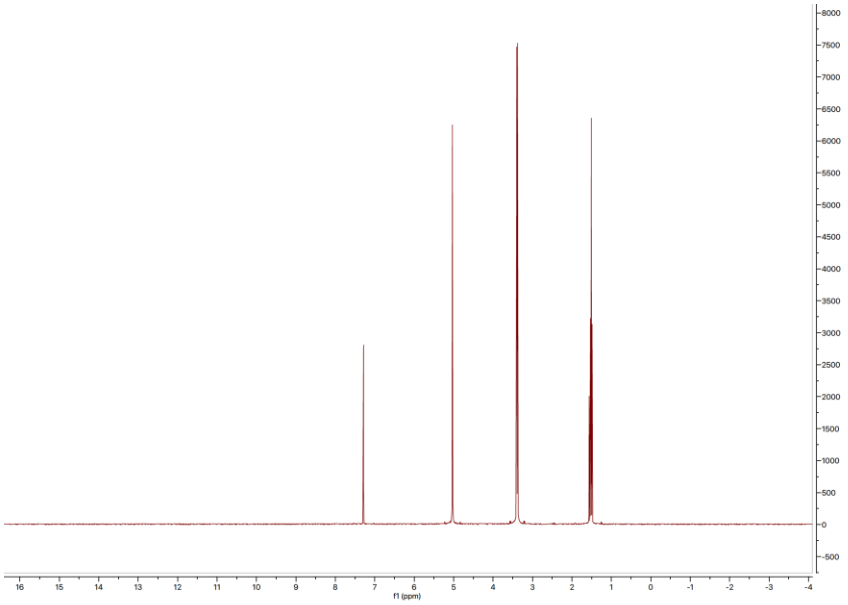

Figure S15. ^1H NMR of allyl dithiol (400 MHz, $CDCl_3$)

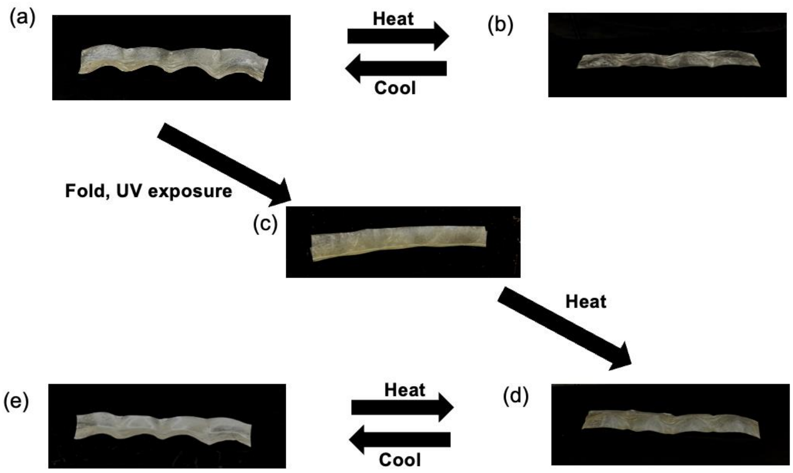

Figure S16. An LCE that has been reprogrammed via dynamic bond exchange (a) in the cool state after first dynamic bond programming, (b) the actuated state (120°C), (c) when folded and exposed to UV light without adding more photoinitiator, (d) when heated after UV exposure, showing little retention of the programmed fold, and (e) after cooling, showing that the LCE returns to the same shape as after the initial dynamic bond programming and undergoes reversible actuation to the behavior before the first programming

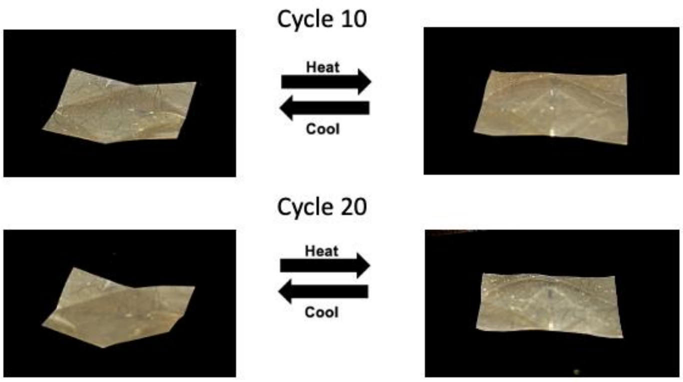

Figure S17. Actuation of the LCE programmed by folding the corners of a +1 topological defects and inducing dynamic bond exchange. Deformations are shown for the LCE after 10 and 20 cycles of heating and cooling, showing little difference in actuation performance upon cycling of the material.

Supporting Videos

Video S1: Reversible actuation of single +1 defect from initial state

Video S2: Reversible actuation of single +1 defect from CAN programmed state

Video S3: Reversible actuation of four adjacent +1 defects from initial state

Video S4: Reversible actuation of four adjacent +1 defects from CAN programmed state

Video S5: Reversible actuation of linearly aligned LCE from initial state

Video S6: Reversible actuation of linearly aligned LCE from 1st CAN programmed state

Video S7: Reversible actuation of linearly aligned LCE from 2nd CAN programmed state

Video S8: linearly aligned LCE without dynamic bonds - actuation from initial state and after folding/light exposure with no secondary programming retention

Video S9: linearly aligned LCE prepared by radical-mediated thiol-acrylate photopolymerization - actuation from initial state and after folding/light exposure with no secondary programming retention
